# Improvement and prediction of the extraction parameters of lupeol and stigmasterol metabolites of *Melia azedarach* with response surface methodology

**DOI:** 10.1186/s12896-024-00865-2

**Published:** 2024-06-07

**Authors:** Vahid Rabbani, Ghasem-Ali Garoosi, Raheem Haddad, Reza Farjaminezhad, Reza Heidari Japelaghi

**Affiliations:** Department of Biotechnology, Faculty of Agriculture and Natural Resources, Imam Khomeini International University (IKIU), P. O. Box 288, Qazvin, l34149-16818 Islamic Republic of Iran

**Keywords:** Box-behnken design, High performance liquid chromatography, Persian lilac, Temperature, Ultrasound-assisted extraction

## Abstract

**Background:**

*Melia azedarach* is known as a medicinal plant that has wide biological activities such as analgesic, antibacterial, and antifungal effects and is used to treat a wide range of diseases such as diarrhea, malaria, and various skin diseases. However, optimizing the extraction of valuable secondary metabolites of *M. azedarach* using alternative extraction methods has not been investigated. This research aims to develop an effective, fast, and environmentally friendly extraction method using Ultrasound-assisted extraction, methanol and temperature to optimize the extraction of two secondary metabolites, lupeol and stigmasterol, from young roots of *M. azedarach* using the response surface methodology.

**Methods:**

Box-behnken design was applied to optimize different factors (solvent, temperature, and ultrasonication time). The amounts of lupeol and stigmasterol in the root of *M. azedarach* were detected by the HPLC-DAD. The required time for the analysis of each sample by the HPLC-DAD system was considered to be 8 min.

**Results:**

The results indicated that the highest amount of lupeol (7.82 mg/g DW) and stigmasterol (6.76 mg/g DW) was obtained using 50% methanol at 45 °C and ultrasonication for 30 min, and 50% methanol in 35 °C, and ultrasonication for 30 min, respectively. Using the response surface methodology, the predicted conditions for lupeol and stigmasterol from root of *M. azedarach* were as follows; lupeol: 100% methanol, temperature 45 °C and ultrasonication time 40 min (14.540 mg/g DW) and stigmasterol 43.75% methanol, temperature 34.4 °C and ultrasonication time 25.3 min (5.832 mg/g DW).

**Conclusions:**

The results showed that the amount of secondary metabolites lupeol and stigmasterol in the root of *M. azedarach* could be improved by optimizing the extraction process utilizing response surface methodology.

**Supplementary Information:**

The online version contains supplementary material available at 10.1186/s12896-024-00865-2.

## Background

Secondary metabolites (SMs), also known as natural products, are organic compounds with low molecular weight, diverse chemical structure, and biological activity that are produced by many plants, bacteria, and fungi. Unlike primary metabolites, these compounds are not necessary for maintaining the organism’s life cycle, reproduction, and growth, but they play an essential role in adaptation to the environment [1]. At present, these compounds have wide applications in various fields including agriculture, pharmaceuticals and cosmetics, farming, and food. In addition, secondary metabolites in non-medical fields such as agriculture in the composition of herbicides, insecticides, and environmentally friendly herbicides and pesticides, and also other industrial products such as beverages, adhesives, and nutraceuticals are being used [2–5]. *Melia azedarach* L. belonging to family Meliaceae is an important medicinal plant in Iran and neighboring countries, which is also known as Chinaberry or Persian Lilac [6]. Moreover, this plant can be found in other regions such as Africa, North America, South America and the south of Europe [7]. In traditional medicine, fruits, flowers, root, seeds and leaves of *M. azedarach* are used to treat a wide range of diseases such as diarrhea, malaria, Various skin diseases [8], chronic intestinal obstruction, purulent sores and treatment of leprosy [9, 10]. Previous studies have shown that organic compounds such as flavonoids, limonoids, tetraterpenoids, organic acids and steroids are abundantly found in *M. azedarach* [7, 11]. The most important secondary metabolites reported in *M. azedarach* are β-sitosterol, squalene, and stigmasterol in the leaves [7], lupeol, β-sitosterol, vanillin, and cyanic acid in the fruit [11], and vanillic acid in the root [7].

The lupeol is a pentacyclic teriterpenoid that is usually found in *M. azedarach* [11, 12]. It has been shown in various studies that lupeol has several important biological activities including anticancer, anti-inflammatory, antiulcerogenic chemopreventive, and antimicrobial properties [12, 13]. The composition of stigmasterol is widely used in the pharmaceutical industry [14]. This compound plays a role in many activities such as anti-osteoarthritis effects [15], anti-inflammatory effects, immunomodulatory effects, neuroprotective effects [14], antibacterial activity [16], antioxidant activity [17], and anticancer properties [14].

Use of different extraction methods to produce secondary metabolites leads to the production of various amounts of these compounds [18]. Simple changes, as changing physical factors or the composition of the extraction solvent will affect the amount of production of secondary metabolites, which this change can be detected and studied [19]. Therefore, identification of the appropriate extraction protocols for the production of secondary metabolites plays an essential role in obtaining high amounts of these compounds.

Industrial and academic sectors are joining other efforts to compare the strengths and weaknesses of innovative and conventional extraction methods in order to choose the most effective method for optimal extraction of target compounds. The general opinion is that there is no universal pattern to choose extraction methods and conditions, therefore, they should be specifically optimized concerning the target composition and matrix of interest [20].

Several methods for the extraction of secondary metabolites have been developed in recent decades, which are mainly divided into two groups’ conventional and new high-technology methods. Conventional methods include the use of organic solvents such as ethanol, methanol, acetone, etc., and/or water, which are usually performed at room temperature. These methods allow soluble metabolites which excreted during growth process and then dissolve in the solvent [21]. Ultrasound-assisted extraction (UAE), pressurized liquid extraction (PLE), microwave-assisted extraction (MAE), etc., are new methods used to extract metabolites. Reducing the time of metabolite extraction, improving performance, and also reducing the volume of solvent used are the advantages of these methods compared to conventional methods [22]. Furthermore, to achieve the maximum amount of secondary metabolites during extraction, parameters such as physical factors (temperature, pH, light, etc.), media components, plant growth regulators, ultrasound time, and type and concentration of the used solvent play important roles [23, 24].

The UAE is an eco-friendly, efficient, and rapid extraction method. This method has the potential to reduce or eliminate the need for organic solvents, thus reducing the negative impact of these solvents on the environment. In addition, UAE significantly increases the production of target secondary metabolites, which makes UAE an attractive method in the industry. However, optimizing the extraction of secondary metabolites using this method requires further investigations, as the current knowledge in this field is limited. Extending the duration of ultrasound exposure can improve the extraction performance of secondary metabolites. However, using of longer ultrasound times can bring the risk of degrading plant metabolites [25].

In addition to the extraction method used, the extraction efficiency and biological activity of the target metabolite are also affected by the extraction solvent [26]. In various studies, solutions such as water, ethanol, methanol, acetonitrile, and acetone have been used to extract secondary metabolites from plant materials [27–29]. Considering the high diversity of secondary metabolites in plant materials and their different solubility properties in diverse solvents, the choice of the appropriate solvent for extraction depends on the type of plant material and the target metabolite. Therefore, the proposition of a suitable solvent for extracting target metabolites from plant materials is generally difficult [26]. Methanol is a very widely used polar solvent for extracting secondary metabolites from plant materials because this solvent can also extract non-polar components in addition to extracting polar components [30].

The temperature of the solvent employed has a significant effect on the extraction of bioactive compounds and must be maintained at its optimum value. When the temperature increases, the kinetic energy of the bioactive compounds also increases. Increased kinetic energy advances the diffusion rate of bioactive compounds into the solvent, allowing for a more efficient extraction process. However, high temperatures can have damaging effects on the integrity of extracted compounds. While high temperature can increase extraction efficiency, there is a threshold beyond which it may lead to reduction or decomposition of extracted compounds [31].

The surface response methodology (RSM) is a very useful statistical approach and a technique to optimize different parameters to find the relation between the factors, as well as the best method of combination of parameters and predicting responses [32]. This method was introduced by Box and Wilson in 1951 [33, 34] and has been enormously used by many researchers. Response Surface Methodology technique helps to solve the problems associated with traditional optimization methods for extraction of metabolites. It helps to save time and money by reducing the number of experimental tests performed [34, 35]. At present, RSM has been successfully used to optimize different variables for extraction of secondary metabolites from cell suspension culture [36] and fungi culture [32], extraction of phenolic compounds and anti-radical activity of *Clinacanthus nutans* Lindau leaves [37].

Zhang et al. used the UAE to extract polyphenolic compounds from *Pinus elliottii* needles. Their results showed that UAE is an effective method for extracting natural polyphenolic compounds from pine needles [38]. In research, optimization of parameters of time, ethanol: water concentration, temperature, and UAE conditions on the content of 2, 2-diphenyl-1-picrylhydrazyl (DPPH), 2, 2′-azino-bis (3-ethylbenzothiazoline-6-sulfonic acid) (ABTS), total phenolic content (TPC) and total flavonoid content (TFC) by RSM in the *Alpinia officinarum* studied. The results revealed that all parameters used had a significant effect (*p* ≤ 0.05) on TPC, DPPH, ABTS, and TFC [39]. Letchumanan et al. optimized the triterpenoid saponins content in *Azadirachta excelsa* leaves by using the RSM and selected independent variables such as ethanol-to-chloroform ratio, temperature, sample-to-solvent ratio, and time [40].

In the present research, a powerful, environmentally friendly, and sensitive method for the extraction of lupeol and stigmasterol metabolites from the root of *M. azedarach* plant using the RSM is reported. To our knowledge, determining the amount of lupeol and stigmasterol metabolites in *M. azedarach* root using RSM under different parameters such as temperature, ultrasonication time, and extraction solvent is reported for the first time.

## Methods and materials

### Chemicals

All chemicals and reagents applied in this research were under analytical grade. Methanol (HPLC grade, 99.9% v/v) and acetonitrile (HPLC grade, 99.5% v/v) solvents were purchased from Merck (Germany), and lupeol (purity ≥ 94%) and stigmasterol (purity ~ 95%) standards were purchased from Sigma-Aldrich (USA).

### Plant material

**Fig. 1:**
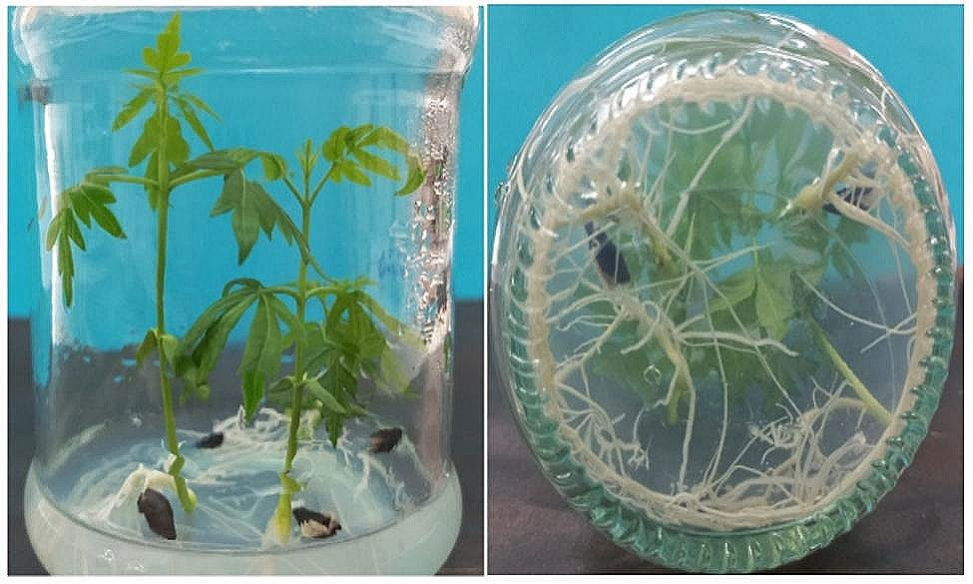
In vitro culture of *M. azedarach*

The seeds of *M. azedarach* were collected from the research field of Imam Khomeini International University (36°19’17.5"N 50°00’42.4"E) and used for cultivation in vitro. First, the seeds were laundered with tap water for 30 min, then disinfected with ethanol (70% v/v) for 50 s and washed with sterile distilled water three times. In the next step, the seeds were disinfected with sodium hypochlorite (2.5% w/v) for 15 min and washed three times with sterile distilled water. Then the sterilized seeds were placed in murashige and skoog medium [41] and incubated at 25 ± 2 °C, 60% relative humidity, with 16 h light and 8 h dark photoperiod and whit 75 µmol m^− 2^ s^− 1^ light intensity. Four-week- old roots were used for extraction of secondary metabolites (Fig. 1). Roots were separated from the plant and washed with tap water for 5 min. The fresh roots of *M. azedarach* dried completely under 35 °C. After the drying process was complete, the dried roots were finely powdered using a mortar and pestle. The powdered roots were screened using a laboratory sieve to ensure uniform particle size, and then the samples were stored in a dry and cool place.

### Preparation of standard solution

The stock solution of Lupeol and stigmasterol as standard solution were prepared seperately by dissolving 0.5 mg of lupeol and stigmasterol standards in 0.5 ml of methanol in a 1.5 ml tube. To completely dissolve lupeol and stigmasterol, the contents of the tubes were sonicated in an ultrasound bath (Elmasonic E30H, 37 kHz, 320 W, Germany) for 5 and 20 min, respectively. In the next step, a set of standard solutions (5, 10, 25, 50, 100, 250, 500 and 1000 µg/ml) was prepared using the dilution series by methanol [42, 43].

### Lupeol and stigmasterol extraction

About 100 mg of powdered root tissue was accurately weighed and added into 2 ml tubes containing 1.5 mL of different concentrations of methanol as a solvent (0, 25%, 50%, 75%, and 100%) and sonicated for 10, 20, 30, 40 and 50 min at various temperatures (25 °C, 30 °C, 35 °C, 40 °C and 45 °C) by ultrasound bath system (Elmasonic E30H, 37 kHz, 320 W, Germany). The obtained mixture was centrifuged at 10,000 rpm for 12 min, and the supernatant was collected. The extracted samples were stored at -20 °C for HPLC-DAD analysis.

### Measurement of lupeol and stigmasterol

The amount of lupeol and stigmasterol in each sample was determined by the Knauer HPLC-DAD system. The detection wavelength for lupeol (Sigma-Aldrich, USA) and stigmasterol (Sigma-Aldrich, USA) were set at 210 nm. The time required for the analysis of each sample by the HPLC-DAD system was considered to be 8 min. The mobile phase used in the present study to determine the simultaneous quantity of lupeol and stigmasterol in the applied treatments was acetonitrile and water (90:10, v/v). After preparing the mobile phase, this phase was degassed in an ultrasound bath for 30 min. A Tosoh C-18 column (TSKgel-ODS C-18, 5 μm, 4.6 × 250 mm, Japan) was used for the separation of the target compound. Also, the flow rate in isocratic mode was 0.9 mL/min. The 20 µl of each sample using a special needle was injected into the HPLC-DAD system. All experiments were performed at room temperature.

### Measurement of lupeol and stigmasterol accumulation in samples

Chromatograms and data of the used samples were recorded using Clarity 7.4.2 software. The accumulation of lupeol and stigmasterol was measured by comparing the standard curve against the peak area (Sigma-Aldrich, USA), and data were reported as mg/g DW.

### Statistical analysis

The RSM method was used to optimize the extraction parameters for lupeol and stigmasterol extraction using Design-expert v.13 software (Stat-Ease, Minneapolis, USA). The box-Behnken Design (BBD) method from RSM was employed to investigate and validate the extraction parameters affecting the extraction efficiency of lupeol and stigmasterol of *M. azedarach* root extracts. According to the obtained results, a second-order polynomial equation was established to predict suitable optimal conditions and comprehend the effect of extraction parameters on the amount of lupeol and stigmasterol extracted. The model for predicted response and response analysis was shown in Equation below:

1 $${\rm{Y}} = {\rm{ }}{\beta _0} + {\rm{ }}\Sigma {\rm{ }}\,{\beta _{\rm{i}}}\,{{\rm{x}}_{\rm{i}}} + {\rm{ }}\Sigma {\rm{ }}\,{\beta _{{\rm{ij}}}}\,{{\rm{x}}^{\rm{2}}}_{\rm{i}} + {\rm{ }}\Sigma {\rm{ }}\,\Sigma {\rm{ }}\,{\beta _{{\rm{ij}}}}\,{{\rm{x}}_{\rm{i}}}\,{{\rm{x}}_{\rm{j}}}$$

In this model, Y is the predicted response, x_i_, and x_j_ represent the independent variables, β_0_, β_i_, β_ii_, β_ij_, and k were model constants, Linear coefficients, Cross product coefficient, quadratic coefficient, and the number of tested variables, respectively. Analysis of variance (ANOVA) was used for the statistical analysis of obtained results at *p* = 0.05 probability level. The model efficiency was studied by the model *p*-value and the coefficient of determination (R^2^).

## Results

### Model fit to predict the amount of lupeol

**Fig. 2:**
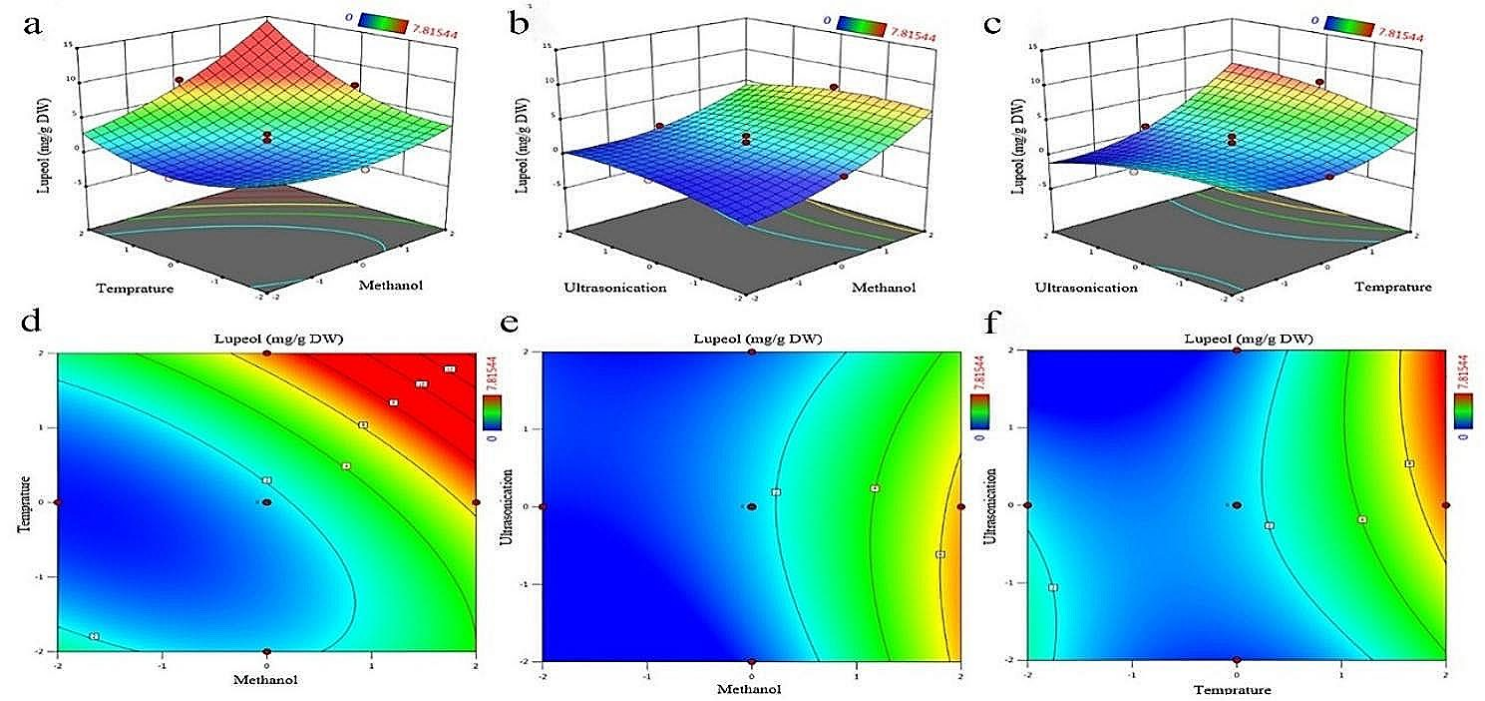
Response surface plots (**a**, **b**, and **c**) and contour plots (**d**, **e**, and **f**) of different factors on the amount of lupeol

According to the results of variance analysis and surface response plots, it was apperceived that solvent concentration and temperature (*p* < 0.05) are the most important effective parameters in the amount of lupeol extracted. The negative result of quadratic terms of ultrasonication time at higher times can be seen in plots b, c, e and f (Fig. 2). Therefore, the optimal conditions for extraction of the maximum amount of lupeol were as follows: 100% methanol, temperature, 45 °C and ultrasonication time, 40 min. According to the experiments conducted in this research, the predicted parameters in experiment 2 (solvent 50% methanol, temperature 45 °C and ultrasonication time 30 min) showed the highest amount of lupeol extraction. In addition, the predicted parameters in experiment 6 (water solvent, temperature of 35 °C, and ultrasonication time of 30 min) showed the lowest amount of lupeol extraction (Table 1). The variance analysis for the optimized equation is indicated in Table 2. According to Table 2, the *p*-value and R^2^ for the model were < 0.0001 and 0.9245, respectively, and the *p*-value for lack of fit was 0.3394. The *p*-value was statistically significant for the model (*p* < 0.01) and non-significant for the lack of fit (*p* = 0.3394). These results indicated that this model is appropriate for prediction of the lupeol amount in the tested range. In this model, the linear parameters x_1_ and x_2_ were statistically significant (*p* < 0.01) and positive, and linear parameter x_3_ was not statistically significant. On the other hand, the interaction parameters x_1_ × _2_ and x_2_ × _3_ were significant (*p* < 0.01 and 0.05 ≤ *p* < 0.1, respectively) while the other parameter (x_1_ × _3_) was not statistically significant. Quadratic parameters x_1_^2^ and x_2_^2^ were positive and significant statistically (0.01 ≤ *p* < 0.05 and *p* < 0.01, respectively), whereas quadratic parameters x_3_^2^ were negative and not statistically significant (Table 3).

**Table 1:** Box–behnken experimental design matrix with experimental responses and predicted values for the amount of lupeol

Run		X_1−_Solvent concentration	X_2−_Temperature (°C)	X_3−_Ultrasonication time (min)	Experimental lupeol (mg/g DW)	Predicted lupeol (mg/g DW)
1		75% Methanol (+ 1)	40 (+ 1)	40 (+ 1)	5.77	6.28
2		50% Methanol (0)	45 (+ 2)	30 (0)	**7.82**	**6.95**
3		25% Methanol (-1)	40 (+ 1)	40 (+ 1)	1.74	2.39
4		75% Methanol (+ 1)	30 (-1)	40 (+ 1)	1.07	1.39
5		50% Methanol (0)	25 (-2)	30 (0)	1.26	1.73
6		Water (-2)	35 (0)	30 (0)	0.0000	0.1495
7		50% Methanol (0)	35 (0)	30 (0)	1.84	1.62
8		50% Methanol (0)	35 (0)	30 (0)	0.8672	1.62
9		75% Methanol (+ 1)	30 (-1)	20 (-1)	3.05	2.80
10		25% Methanol (-1)	30 (-1)	20 (-1)	0.4892	0.3761
11		50% Methanol (0)	35 (0)	50 (+ 2)	1.01	0.7266
12		100% Methanol (+ 2)	35 (0)	30 (0)	**7.01**	**6.46**
13		50% Methanol (0)	35 (0)	10 (-2)	0.5605	0.4505
14		50% Methanol (0)	35 (0)	30 (0)	1.17	1.62
15		75% Methanol (+ 1)	40 (+ 1)	20 (-1)	4.79	5.70
16		50% Methanol (0)	35 (0)	30 (0)	2.81	1.62
17		25% Methanol (-1)	30 (-1)	40 (+ 1)	0.5837	0.0705
18		50% Methanol (0)	35 (0)	30 (0)	1.57	1.62
19		50% Methanol (0)	35 (0)	30 (0)	1.85	1.62
20		25% Methanol (-1)	40 (+ 1)	20 (-1)	0.6295	0.7030

**Table 2:** Analyses of variance for the response surface quadratic model to optimize extraction parameters of lupeol and stigmasterol

Source	Stigmasterol (*R*^2^ = 0.9245)						Lupeol (*R*^2^ = 0.9245)				
	SS	df	MS	F-value	*p*-value		SS	df	MS	F-value	*p*-value
Model	91.27	9	10.14	13.61	0.0002		92.21	9	10.25	18.19	< 0.0001
x_1_-Solvent	5.24	1	5.24	7.04	0.0242		39.86	1	39.86	70.76	< 0.0001
x_2_-Temperature	0.1351	1	0.1351	0.1814	0.6792		27.18	1	27.18	48.26	< 0.0001
x_3_-Ultrasonication time	2.33	1	2.33	3.13	0.1073		0.0762	1	0.0762	0.1353	0.7207
x_1_ x_2_	5.53	1	5.53	7.42	0.0214		3.30	1	3.30	5.86	0.0361
x_1_ x_3_	0.1219	1	0.1219	0.1637	0.6943		0.6098	1	0.6098	1.08	0.3226
x_2_ x_3_	19.20	1	19.20	25.78	0.0005		1.98	1	1.98	3.52	0.0900
x_1_^2^	33.96	1	33.96	45.59	< 0.0001		4.48	1	4.48	7.95	0.0182
x_2_^2^	36.32	1	36.32	48.75	< 0.0001		11.63	1	11.63	20.65	0.0011
x_3_^2^	1.48	1	1.48	1.98	0.1892		1.67	1	1.67	2.96	0.1162
Residua	7.45	10	0.7449				5.63	10	0.5633		
Lack of fit	1.64	5	0.3277	0.2819	0.9045		3.36	5	0.6719	1.48	0.3394
Pure error	5.81	5	1.16				2.27	5	04547		
Cor (Total)	98.72	19					97.84	19			

**Table 3:** Estimated coefficients for the fitted second-order polynomial model for the amount of lupeol and stigmasterol

Term	Regression coefficients		
	Stigmasterol		Lupeol
Intercept	5.69		1.62
Linear			
β1	-0.5724**		1.58*
β2	-0.0919		1.30*
β3	-0.3817		0.0690
Interaction			
β12	0.8318**		0.6421**
β13	0.1235		-0.2761
β23	-1.55*		0.4979***
Quadratic			
β11	-1.16*		0.4220**
β22	-1.20*		0.6802*
β33	-0.2425		-0.2572

* *p* < 0.01, ** 0.01 ≤ *p* < 0.05, and *** 0.05 ≤ *p* < 0.1

Finally, the optimal conditions for description of the efficiency of lupeol extraction were predicted and designed as the following Equation:

2 $$\eqalign{{\rm{y}}\,{\rm{ = }}\, & {\rm{1}}{\rm{.62 + 1}}{\rm{.58}}\,{{\rm{x}}_{\rm{1}}}{\rm{ + 1}}{\rm{.30}}\,{{\rm{x}}_{\rm{2}}}{\rm{ + 0}}{\rm{.0690}}\,{{\rm{x}}_{\rm{3}}} \cr & {\rm{ + 0}}{\rm{.6421}}\,{{\rm{x}}_{\rm{1}}}{{\rm{x}}_{\rm{2}}}{\rm{ - 0}}{\rm{.2761}}\,{{\rm{x}}_{\rm{1}}}{{\rm{x}}_{\rm{3}}}{\rm{ + 0}}{\rm{.4979}}\,{{\rm{x}}_{\rm{2}}}{{\rm{x}}_{\rm{3}}} \cr & {\rm{- 0}}{\rm{.4220}}\,{{\rm{x}}_{\rm{1}}}^{\rm{2}}{\rm{ + 0}}{\rm{.6802}}\,{{\rm{x}}_{\rm{2}}}^{\rm{2}}{\rm{ - 0}}{\rm{.2574}}\,{{\rm{x}}_{\rm{3}}}^{\rm{2}} \cr}$$

### Response surface analysis for the amount of lupeol

Table 1 indicates the factors affecting lupeol extraction. Figure 2 was drawn according to Eq. (1). The highest amount of lupeol in this research was obtained at the maximum temperature, the highest concentration of methanol solution, and an ultrasonication time of 40 min. These results show that high concentrations of methanol as well as high temperatures have a great effect on the extraction of lupeol and the increase of these two parameters leads to an increase in the amount of lupeol. In addition, increase of the ultrasonication time up to 40 min enhanced the amount of lupeol and then, with increase of time, the amount of lupeol decreased.

### Model fit to predict the amount of stigmasterol

Table 4 shows the code of the performed tests and the actual and predicted values for stigmasterol. The actual values for stigmasterol varied from 0 mg/g DW to 6.76 mg/g DW, and the predicted values for stigmasterol from 0 mg/g DW to 5.69 mg/g DW. According to the data in Table 4, experiment 19 (50% methanol, temperature 30 °C, and ultrasonication time 30 min) showed the highest amount of stigmasterol (6.76 mg/g DW). While experiment 9 (75% methanol, temperature 30 °C, and ultrasonication time 30 min) provided the lowest amount of stigmasterol (0.00 mg/g DW). The variance analysis has indicated in Table 2. The *p*-value and R^2^ for the model were 0.0002 and 0.9245, respectively, and the *p*-value for lack of fit was 0.9045. The *p*-value was statistically significant for the model (*p* < 0.01) and non-significant for the lack of fit (*p* = 0.9045). According to these results, it can be suggested that this model is appropriate for prediction of the amount of stigmasterol in the tested range. In this model, the linear parameter x_1_ was negative and statistically significant (0.01 ≤ *p* < 0.05), whereas linear parameters x_2_ and x_3_ were not statistically significant and negative. On the other hand, the interaction between parameters x_1_ × _2_ and x_2_ × _3_ were statistically significant (0.01 ≤ *p* < 0.05 and *p* < 0.01, respectively) and the other parameter (x_1_ × _3_) was positive and not statistically significant. Quadratic parameters x_1_^2^ and x_2_^2^ were negative and significant statistically (*p* < 0.01 and *p* < 0.01, respectively), whereas quadratic parameter x_3_^2^ were not statistically significant and negative (Table 3). Finally, the optimal conditions for description of the efficiency of stigmasterol extraction were predicted and designed as the following equation:

3 $$\eqalign{{\rm{y}}\,{\rm{ = }}\, & {\rm{5}}{\rm{.69- 0}}{\rm{.5724}}\,{{\rm{x}}_{\rm{1}}}{\rm{- 0}}{\rm{.0919}}\,{{\rm{x}}_{\rm{2}}}{\rm{- 0}}{\rm{.3817}}\,{{\rm{x}}_{\rm{3}}} \cr & {\rm{ + 0}}{\rm{.8313}}\,{{\rm{x}}_{\rm{1}}}{{\rm{x}}_{\rm{2}}}{\rm{ + 0}}{\rm{.1235}}\,{{\rm{x}}_{\rm{1}}}{{\rm{x}}_{\rm{3}}}{\rm{-1}}{\rm{.55}}\,{{\rm{x}}_{\rm{2}}}{{\rm{x}}_{\rm{3}}} \cr & {\rm{- 1}}{\rm{.16}}\,{{\rm{x}}_{\rm{1}}}^{\rm{2}}{\rm{- 1}}{\rm{.20}}\,{{\rm{x}}_{\rm{2}}}^{\rm{2}}{\rm{- 0}}{\rm{.2425}}\,{{\rm{x}}_{\rm{3}}}^{\rm{2}} \cr}$$

### Response surface analysis for the amount of stigmasterol

**Fig. 3:**
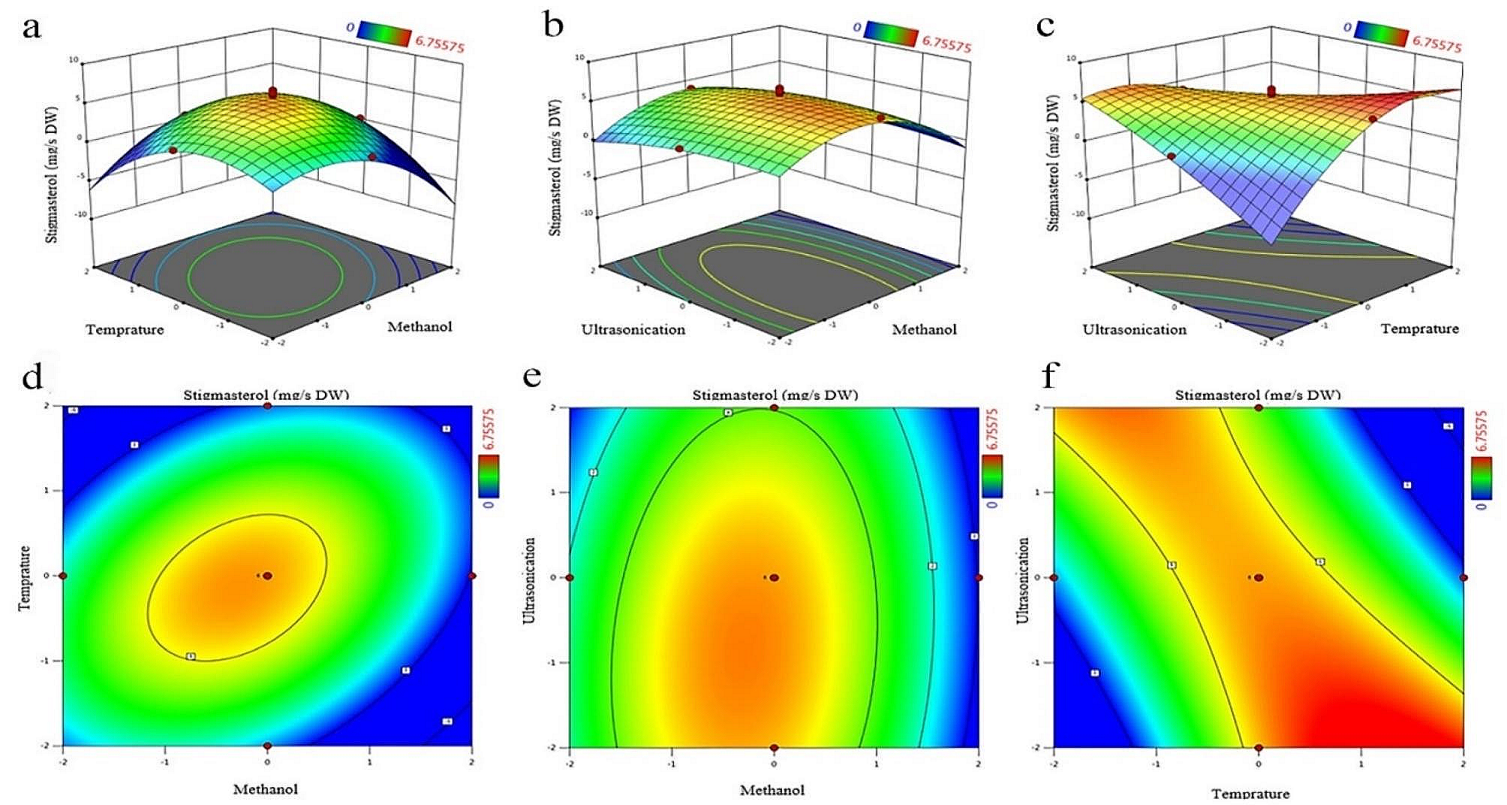
Response surface plots (**a**, **b** and **c**) and contour plots (**d**, **e**, and **f**) of different factors on the amount of stigmasterol

The two-dimensional and three-dimensional schemes shown in Fig. 3 display the relationship between the amount of stigmasterol (dependent variable) and x_1_ as solvent concentration, x_2_ as temperature, and x_2_ as ultrasonication time (independent variables). In this study, the amount of stigmasterol gradually decreased with increase of methanol concentration, temperature, and ultrasonication time. The lowest amount of stigmasterol was observed in 100% methanol, 45 °C, and ultrasonication time of 50 min. A similar effect of solvent on extraction metabolites such as flavonoids has been mentioned in previous studies. This might be because the presence of water in the solvent stimulates the swelling action in the plant matrix and increases the contact surface usable for the solvent [44]. The negative effect of temperature on the amount of stigmasterol can be seen in plots a, c, d, and f Fig. 3. Increase of temperature in low levels of methanol solvent led to an increase in the amount of stigmasterol, but in high concentrations of methanol, increase of the temperature caused a decrease in the amount of stigmasterol. These results showed that the interaction between methanol concentration and temperature at lower levels has a positive effect on the amount of stigmasterol extraction. Therefore, this will prohibit the decomposition of stigmasterol from *M. azedarach*. Figure 3b and e indicate the interaction between different concentrations of methanol and different ultrasonication time on the amount of stigmasterol extraction, which this interaction was statistically significant (0.01 ≤ *p* < 0.05(. The study of the interaction between temperature and ultrasonication time revealed that increasing temperature and decreasing ultrasonication time result in an increase in the amount of stigmasterol, which this increase in the amount of stigmasterol was statistically significant (*p* < 0.01) (Fig. 3c and f). Therefore, the optimal conditions for extraction of the maximum amount of stigmasterol were as follows: 43.75% methanol, temperature, 34.4 °C, and ultrasonication time, 25.3 min.

**Table 4:** Box–behnken experimental design matrix with experimental responses and predicted values for the amount of stigmasterol

Run	X_1_-Solvent concentration	X_2_-Temperature (°C)	X_3_-Ultrasonication time (min)	Experimental stigmasterol (mg/g DW)	Predicted stigmasterol (mg/g DW)
1	75% Methanol (+ 1)	40 (+ 1)	40 (+ 1)	1.45	1.44
2	50% Methanol (0)	45 (+ 2)	30 (0)	0.7295	0.6985
3	25% Methanol (-1)	40 (+ 1)	40 (+ 1)	0.6178	0.6779
4	75% Methanol (+ 1)	30 (-1)	40 (+ 1)	2.67	3.06
5	50% Methanol (0)	25 (-2)	30 (0)	1.58	1.07
6	Water (-2)	35 (0)	30 (0)	2.37	2.19
7	50% Methanol (0)	35 (0)	30 (0)	**6.33**	**5.69**
8	50% Methanol (0)	35 (0)	30 (0)	**6.40**	**5.69**
9	75% Methanol (+ 1)	30 (-1)	20 (-1)	0.0000	0.4803
10	25% Methanol (-1)	30 (-1)	20 (-1)	2.98	3.53
11	50% Methanol (0)	35 (0)	50 (+ 2)	3.97	3.96
12	100% Methanol (+ 2)	35 (0)	30 (0)	0.2565	0
13	50% Methanol (0)	35 (0)	10 (-2)	6.01	5.48
14	50% Methanol (0)	35 (0)	30 (0)	3.82	5.69
15	75% Methanol (+ 1)	40 (+ 1)	20 (-1)	4.66	5.06
16	50% Methanol (0)	35 (0)	30 (0)	5.29	5.69
17	25% Methanol (-1)	30 (-1)	40 (+ 1)	5.48	5.62
18	50% Methanol (0)	35 (0)	30 (0)	6.09	5.69
19	50% Methanol (0)	35 (0)	30 (0)	**6.76**	**5.69**
20	25% Methanol (-1)	40 (+ 1)	20 (-1)	4.64	4.79

### Identification of lupeol and stigmasterol

**Fig. 4:**
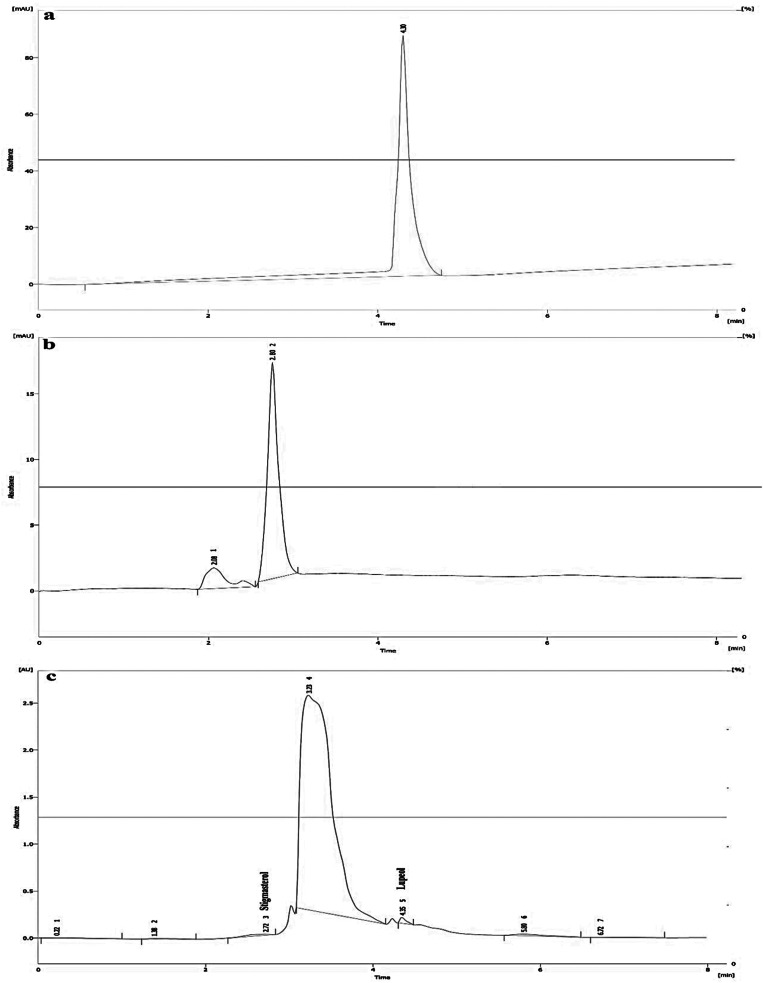
HPLC-DAD chromatograms of the lupeol (**a**), stigmasterol (**b**) and extraction samples (**c**). The retention times for lupeol and stigmasterol standards at 210 nm were 4.30 and 2.8 min, respectively

Figure 4 indicates the HPLC-DAD chromatograms of lupeol and stigmasterol samples and standards. The peaks of lupeol and stigmasterol were identified by HPLC-DAD with retention times 4.30 and 2.80 min, respectively.

## Discussion

In this research, to optimize the extraction of lupeol and stigmasterol metabolites in the root of *M. azedarach* using RSM, three parameters of temperature, ultrasonication time, and solvent concentration were used. In many studies, RSM has been used to optimize the extraction of secondary metabolites using parameters such as extraction temperature, time, ultrasound time, type of solvent, solid-liquid ratio, and use of microwaves [27]. Letchumanan et al. used the RSM to optimize the extraction of Triterpenoid Saponins using parameters such as temperature, time, ethanol-to-chloroform ratio, and sample-to-solvent ratio in *Azadirachta excelsa* [40]. In addition, Farjaminezhad and Garoosi. used the RSM method to optimize the extraction of azadirachtin, mevalonic acid, and squalene metabolites from the cell suspension culture of *Azadirachta indica*. These results showed that RSM can be used to optimize the extraction process of other secondary metabolites in a wide range of plants [45]. In the current study, methanol was used as the solvent of choice for the extraction experiments, considering the high polarity of triterpenoids, polarity index, and dielectric constant of the solvent, as well as previous studies [46]. In the present experiment, the effect of different concentrations of methanol solvent (0, 25, 50, 75, and 100%) on lupeol extraction was significant (Table 2). This result agrees with the study of Sabaragamuwa and Perera [47]. Sabaragamuwa and Perera. investigated the effect of different ratios of methanol and water mixtures on the total triterpene content (TTC) in *Centella asiatica*. Their results revealed that the amount of TTC gradually increased with the increase of methanol percentage in the extraction solvent [47]. Das et al. used the RSM method to identify the effect of variables such as microwave power, irradiation time, variable solvent strength, particle size, solvent, sample ratio, and prewashing time on lupeol extraction in *Ficus racemosa*. Their results showed that the use of different concentrations of methanol (50–100% v/v) had no significant effect (*P* < 0.05) on lupeol extraction, which is not consistent with the findings of the current study [48]. This difference can be attributed to the effect of temperature on the extraction of lupeol. This shows that temperature plays an important role in lupeol production because obvious differences can be seen between different treatments (Table 1). At high temperatures, some triterpenes may be oxidized and thus contain hydroxyl groups [49–51]. Therefore, in this research, to extract lupeol from the root of the *M. azedarach* plant, a relatively mild time and temperature were chosen to reach the maximum amount of metabolite [52].

According to the results of the variance analysis and surface response scheme, it was apperceived that solvent concentration and temperature (*p* < 0.05) are the most importance effective parameters in the amount of lupeol extracted. This shows that both temperature and solvent composition have an important role in extraction efficiency of lupeol. This increase in the extraction yield of triterpenoids was also observed in the study of Pandi and Kaur., which was consistent with the results of the present study. They reported that the maximum yield of triterpenoids in the stem of *Swertia chirata* was observed at 65 °C and a solvent composition of 50% methanol [53]. As the temperature increases, the viscosity of the solvent decreases, which results in an increase in the wetting of the matrix and the solubilization of metabolites. In addition, owing to the increase in temperature, more energy is spent to break the matrix-metabolites bond, and therefore the diffusion of these metabolites in the solvent increases [53]. In other studies, similar results were reported in the extraction of triterpenoids, in which temperature and solvent composition played an important role in increasing the amount of triterpenoids [53, 54].

In the present study, the amount of lupeol enhanced with the increase in ultrasonication time, indicating that UAE can increase the leaching rate of different triterpenes in the solvent and have a similar effect on other secondary metabolites [55]. Schinor et al. in a study, showed that the amount of metabolite obtained using UAE for 30 min was comparable to extraction by maceration method for 24 h. In addition, they reported that the amount of metabolite obtained using UAE was more or equal to the maceration method, which indicates a notable reduction in the extraction time and also an increase in the extraction efficiency [56]. These results show that the extraction speed of metabolites with UAE is several times faster than the conventional method.

In the present study, the predicted conditions (100% methanol, temperature 45 ۫°C, and ultrasonication time 40 min) showed the highest amount of lupeol (14.540 mg/g DW). Macías-Rubalcava et al. investigated the amount of lupeol in different plants and reported 0.003 mg/g in *Olea europaea* fruit, 0.152 mg/g in *Panax ginseng* oil, 0.175 mg/g in *Pyrus pyrifolia* and 0.880 mg/g DW in *Ulmus plant* [57]. Moreover, the amount of lupeol was investigated in the root, green leaf, and fruit of the *Coccoloba uvifera* L. using different extraction techniques. The results showed that the highest amount of lupeol (5.606 mg/g DW) was observed using UAE in green leaves of the *Coccoloba uvifera* L. plant [58]. In all cases, the amounts were inferior to those obtained from the roots of the *M. azedarach* plant (Table 1). In this sense, the root of *M. azedarach* shows a large amount of lupeol.

Ahmad et al. reported the optimization of extraction of a phytosterol (Charantin) using the RSM method and parameters such as methanol (70–100% v/v), temperature (30–60 °C), time (30–90 min) and solid to solvent ratio (1:10–1:25, w/v) in *Momordica charantia* fruit. Their study showed that the content of this phytosterol increases with the increase of water percentage (up to 20%) in methanol solvent [59]. In addition, their results showed that with the increase of methanol percentage from 80 to 100%, the contents of extracted metabolite gradually decreased. Their results are consistent with the present study [59]. The results of the present research show that the combination of water with methanol led to an increase in the amount of stigmasterol obtained at low temperatures, which is very important due to easy access to water, non-flammability, environmentally friendly, and non-toxicity [60]. In addition, methanol is cheap and easily available, so it can be a very suitable solvent in combination with water for extraction of sterols compared to other solvents.

In the present study, an increase in temperature at low levels enhanced the amount of stigmasterol, but high temperatures led to a decrease in the amount of this metabolite. The negative effect of temperature on the amount of stigmasterol can be seen in schemes a, b, c, and d (Fig. 3). These results are consistent with the studies of other researchers [61–63]. Nyam et al. reported that the optimal temperature for the extraction of sterols is between 30 and 42 °C [61]. On the other hand, Tramontin et al. described the best temperature for the extraction of sterols to be 40 °C [63]. This difference in temperature may be related some researches considered different type of sterols, while in the present study, the extraction conditions were evaluated for a single sterol. Alam et al. showed that increasing the temperature from 30 to 60 °C led to a significant enhancement in the extraction efficiency of β-sitosterol metabolite, while with a further increase in temperature to 80 °C, no significant increase in the extraction efficiency of β-sitosterol metabolite was observed [64]. In another study, Basilio-Cortes et al. investigated the effect of temperature on the extraction of secondary metabolites in hops. Their results showed that as the temperature increased from 25 °C to 57.5 °C, total phenol and flavonoid content in hop samples increased. However, with the increase in temperature to 90 °C, the amount of these compounds decreased, which is probably due to their denaturation under high temperature and the duration of exposure to temperature [65].

The results of the present study showed that the amount of stigmasterol enhanced with the increase of ultrasonication time from 10 to 25 min, while no increase in the amount of stigmasterol was observed after this time. This shows that the ultrasonication time affects the liquid circulation and the turbulence produced, which increases the Extraction efficiency by increasing the contact surface between the target compound and the solvent [66]. Alam et al. showed that the efficiency of β-sitosterol metabolite enhanced significantly with increasing time from 10 to 40 min, while after 40 min, the increase in the efficiency of these metabolites was non-significant [64]. These results are consistent with the findings of the present study. In a similar study, Siddiqui and Aeri. investigated the effect of different ultrasound times on the extraction yield of stigmasterol in the bark of *Tecomella undulata*. Their results showed that the amount of this metabolite gradually increased in the range of 35 to 46.86 min, but outside this range, the extraction yield of stigmasterol decreased with the increase in ultrasound time [67]. These results show that the decrease in phytosterol extraction yield at longer ultrasound time can be attributed to the possibility of their decomposition by the effect of sound waves. This is one of the disadvantages of the UAE technique [68].

These results showed that the interaction between methanol concentration and temperature at lower levels has a positive effect on the amount of stigmasterol extraction. Therefore, this will prohibit the decomposition of stigmasterol from *M. azedarach*. The combination of water with methanol leads to a significant decrease in the polarity of water without the need to increase the temperature. In addition, compared to pure water, the mixture of water and methanol forms a solvent with a lower density, which has less hydrogen bonding strength between water molecules, higher diffusivity, and less surface tension [69]. In this way, during the metabolite extraction process, a higher permeability occurs in the cellular structures of the matrix, which increases the extractability of the metabolite [70]. According to the studies, higher temperatures increase the solubility and dispersion of metabolites in the solvent, but the results of the present study showed that high temperatures had a negative effect on the stigmasterol content [71]. These results showed that high temperature degraded this compound like other phytosterols [59]. The decrease in stigmasterol content at high temperatures shows the sensitivity of phytosterols to temperature, which was reported in the study of Siddiqui and Aeri [67]. Farjaminezhad and Garoosi. reported that temperature and methanol concentration have a positive interaction with azadirachtin content. In that study, they showed that the content of azadirachtin decreases with increasing temperature, which is consistent with the results of the present study [45].

## Conclusions

In the current study, for the first time modeling and optimization of extraction of secondary metabolites lupeol and stigmasterol from *M. azedarach* root was successfully performed using response surface methodology. The aim of the optimization was the simultaneous maximization of the extraction yield of lupeol and stigmasterol. BBD was successfully used to improve efficiency and study the effects of extraction factors such as temperature, solvent, and ultrasonication time on the amount of lupeol and stigmasterol. In the present research, the quadratic models illustrated the relevance between the variables and responses. Examination of different mono- and binary-solvent systems based on methanol and water revealed information on the extraction behavior of lupeol and stigmasterol in *M. azedarach* root. The extraction of lupeol and stigmasterol compounds varied with different solvent ratios of the extraction mixture, mainly based on the polarity of the compounds. This investigation showed that a binary-solvent system using a combination of water and methanol led to better extraction of stigmasterol from *M. azedarach* root. In addition, our results showed that high concentrations of methanol and high temperatures have a significant effect on the extraction of lupeol, and the increase of these two parameters leads to an increase in the amount of lupeol. The best conditions for lupeol and stigmasterol extraction from the root of *M. azedarach* were 100% methanol, temperature 45 °C and ultrasonication time 40 min; and 43.75% methanol, temperature 34.4 °C and ultrasonication time 25.3 min, respectively.

### Electronic supplementary material

Below is the link to the electronic supplementary material.

Supplementary Material 1

Supplementary Material 2

## Data Availability

All data generated or analyzed during this study are included in this published article and its supplementary information files.
