# Supplementary material for: Improvement and prediction of the extraction parameters of lupeol and stigmasterol metabolites of Melia azedarach with response surface methodology
Source: BMC Biotechnol. 2024 Jun 7;24:39. doi: 10.1186/s12896-024-00865-2 (PMC11157759; doi:10.1186/s12896-024-00865-2)

**Supplementary 2.** Response surface plots (a, b and c) and contour plots (d, e, and f) of different factors on the amount of stigmasterol.

a)


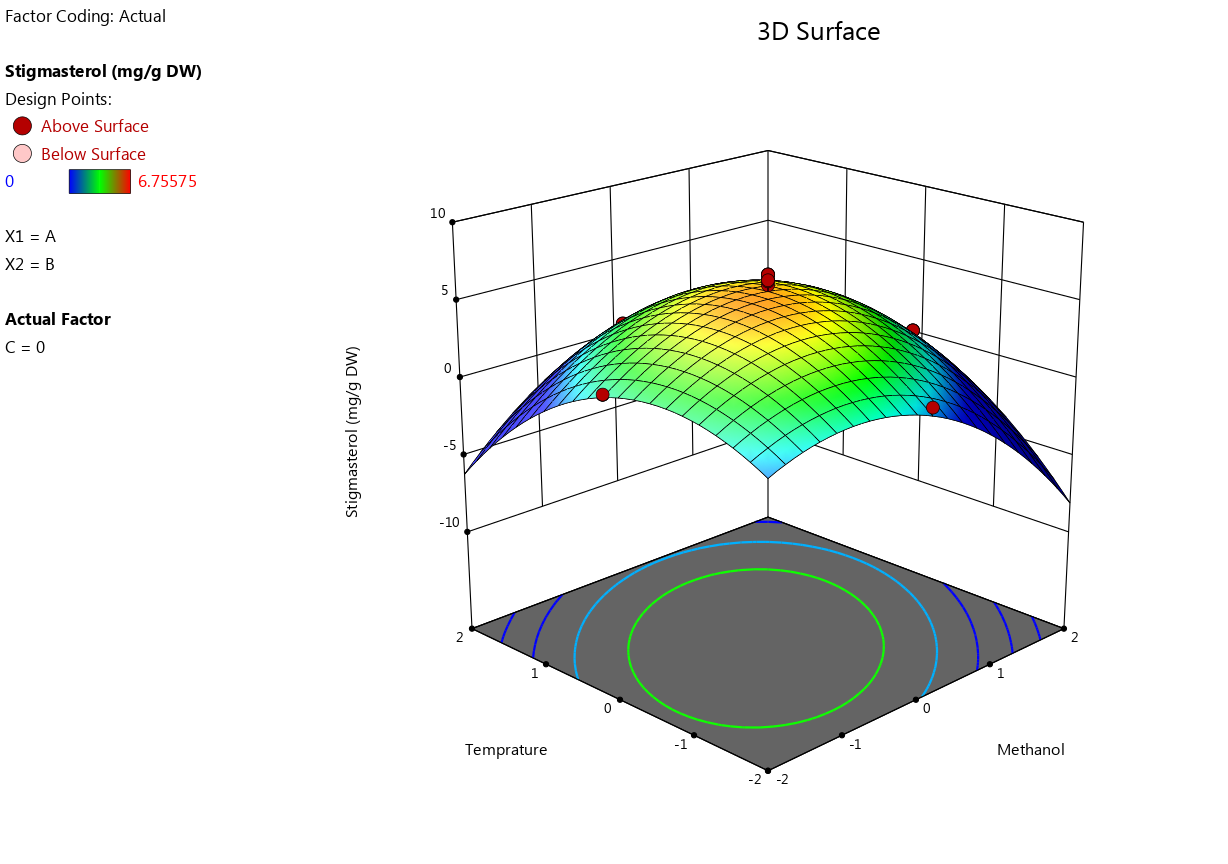


b)


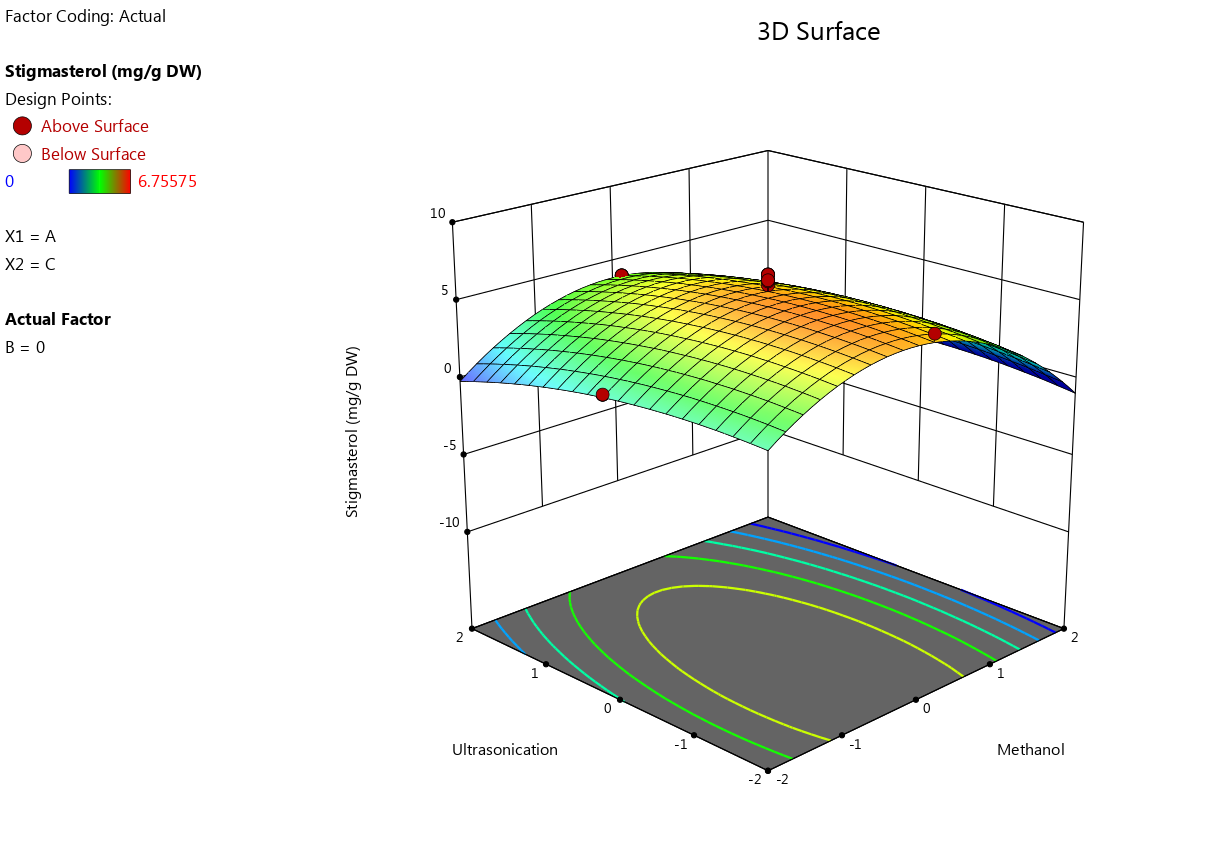


c)


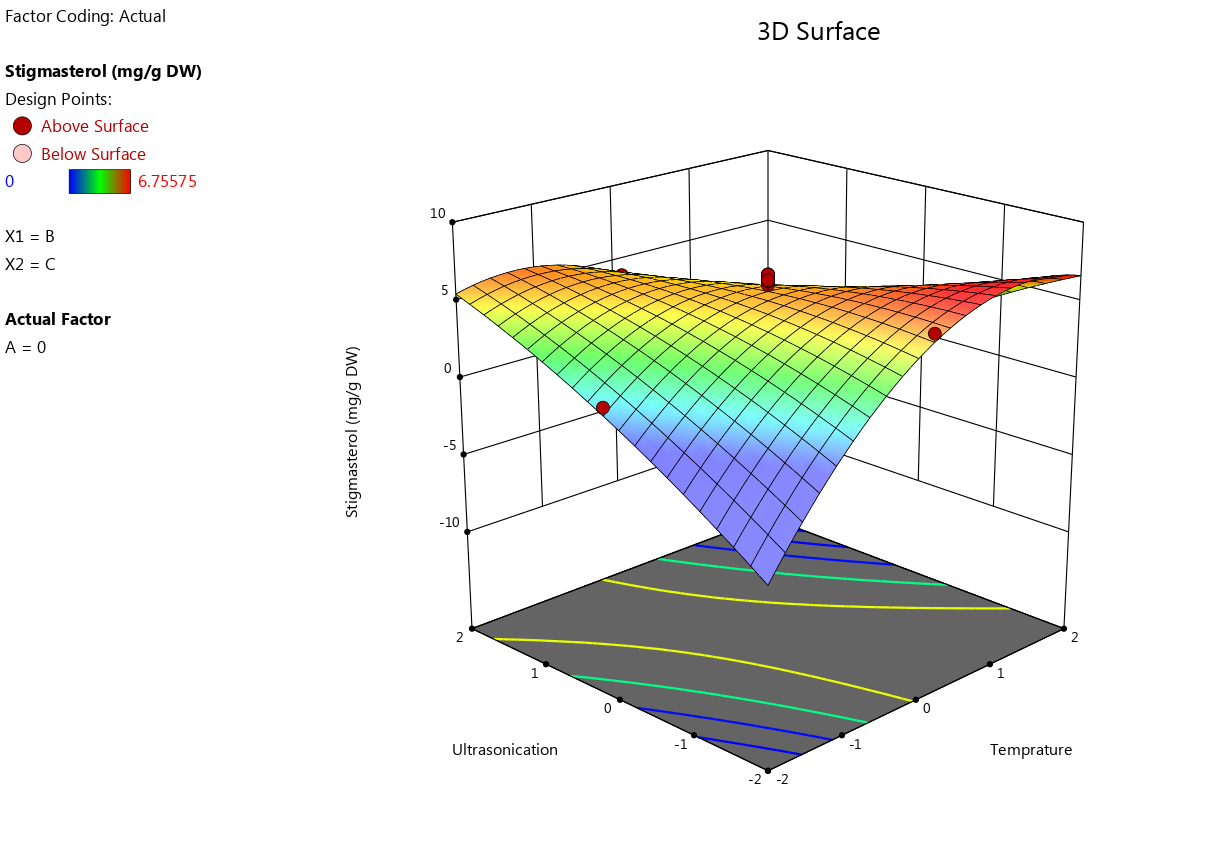


d)


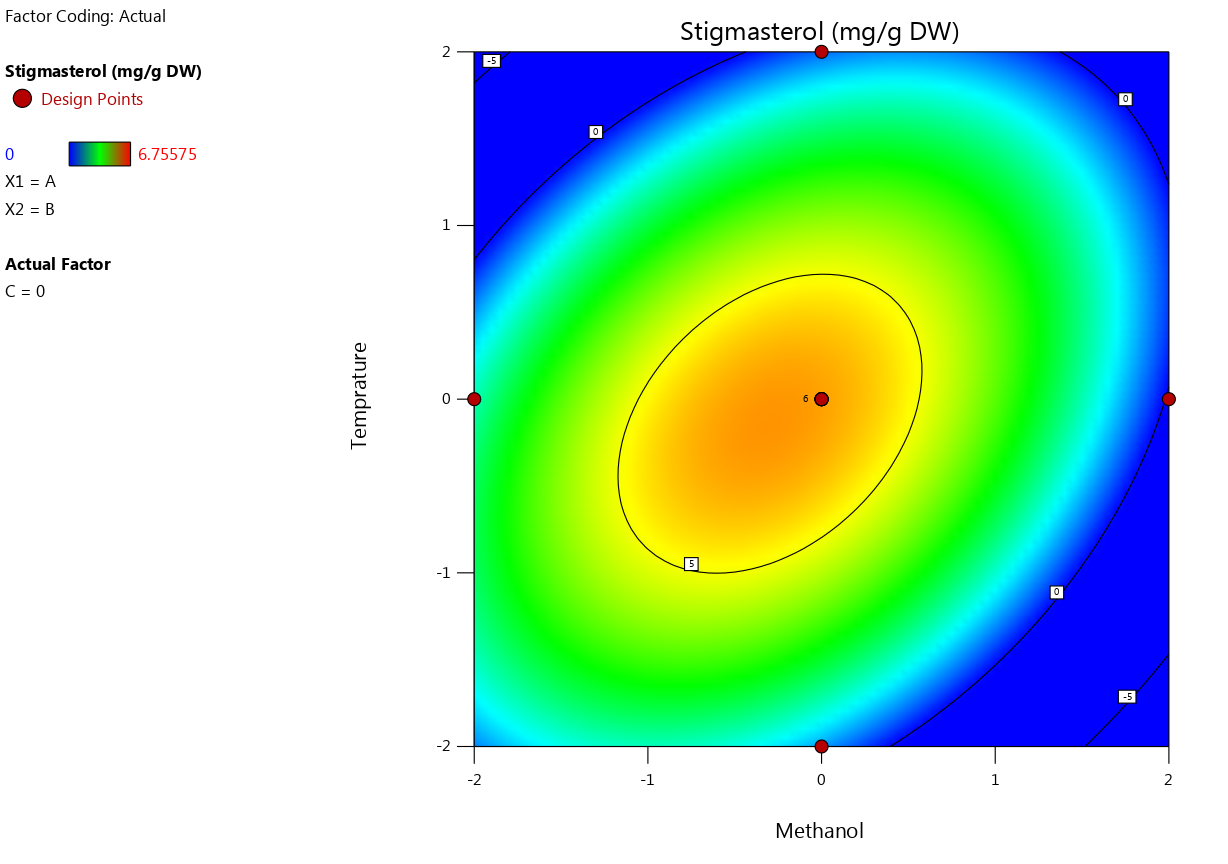


e)


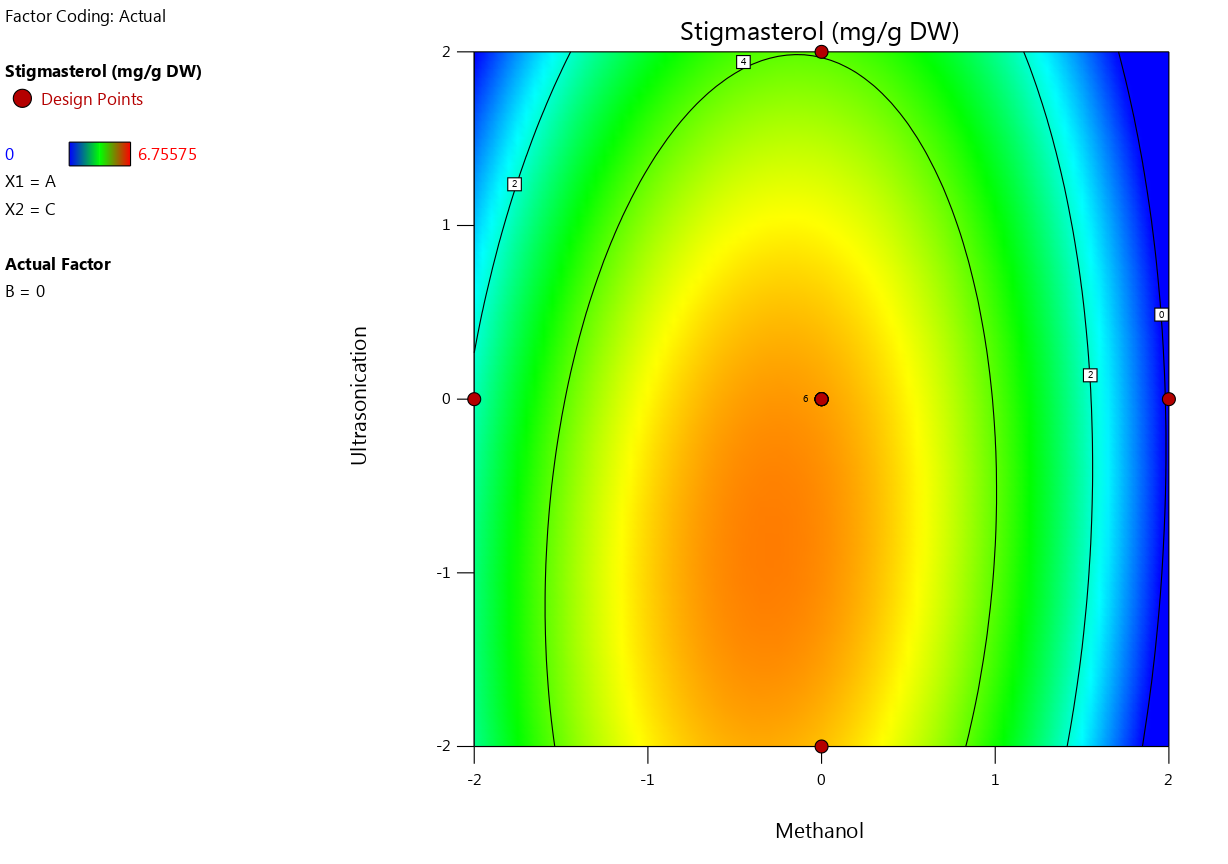


f)


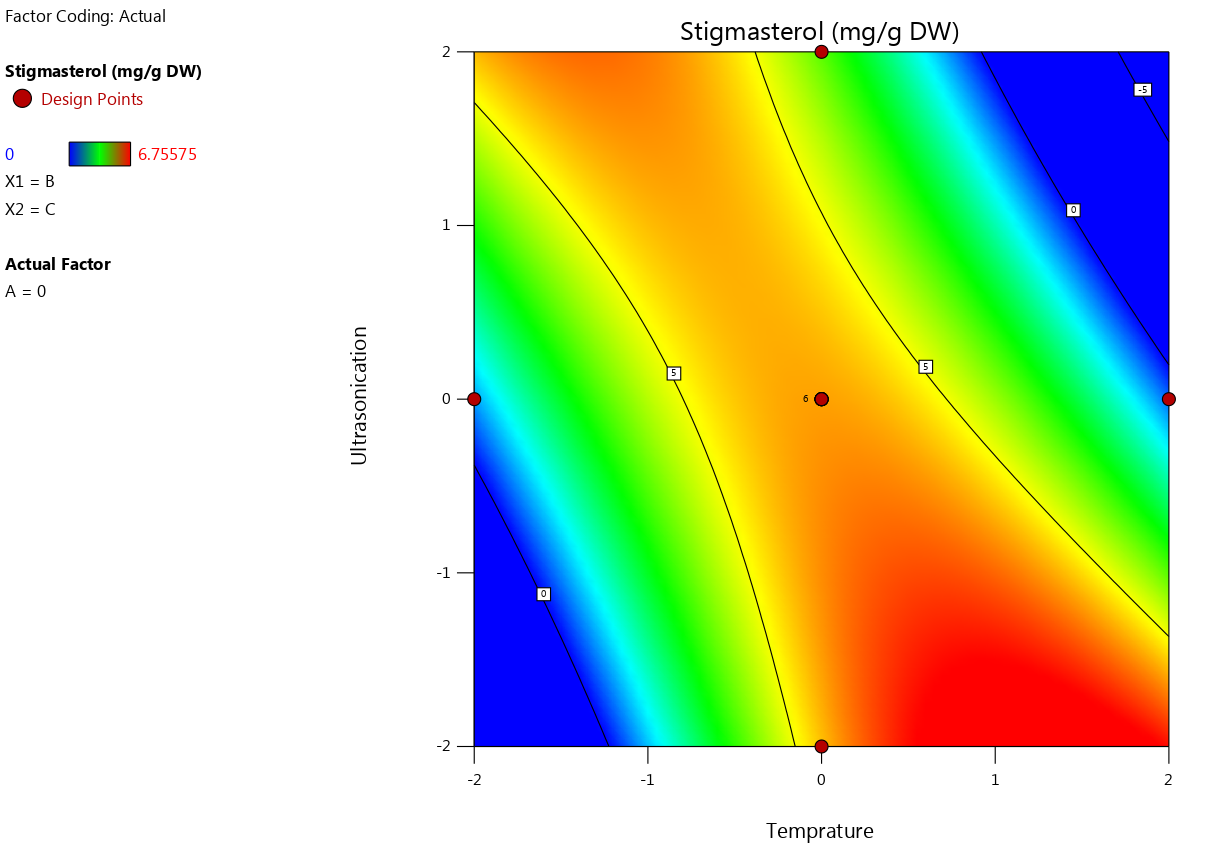

Supplement: Supplementary file 2 — Supplementary Material 2 [file 12896_2024_865_MOESM2_ESM.docx]
